# D-Mannose Slows Glioma Growth by Modulating Myeloperoxidase Activity

**DOI:** 10.3390/cancers13246360

**Published:** 2021-12-18

**Authors:** Negin Jalali Motlagh, Cuihua Wang, Enrico Giovanni Kuellenberg, Gregory R. Wojtkiewicz, Stephan Schmidt, John W. Chen

**Affiliations:** 1 Institute for Innovation in Imaging, Department of Radiology, Massachusetts General Hospital, Harvard Medical School, Charlestown, MA 02129, USA; NJALALIMOTLAGH@mgh.harvard.edu (N.J.M.); Wang.Cuihua@mgh.harvard.edu (C.W.); EKUELLENBERG@mgh.harvard.edu (E.G.K.); 2 Center for Systems Biology, Massachusetts General Hospital, Harvard Medical School, Boston, MA 02114, USA; GWOJTKIEWICZ@mgh.harvard.edu (G.R.W.); SPSCHMIDT@mgh.harvard.edu (S.S.)

**Keywords:** D-mannose, leukocytes, MPO activity, glioma

## Abstract

**Simple Summary:**

Inflammation and oxidative stress are important host defense responses. However, while the host response can be cytotoxic and kill tumor cells, tumor cells can also alter and exploit the host immune environment to further their survival. Thus, the host response can impact both tumor suppression and progression. Modulating the tumor–host response interaction to favor tumor suppression would be highly desirable. D-mannose has been found to have anti-inflammatory properties and can block signaling related to myeloperoxidase (MPO), a highly oxidizing pro-inflammatory enzyme secreted in host defense. However, the effect of D-mannose on host immune response in the glioma microenvironment has not been explored. We found that D-mannose slowed glioma growth by increasing MPO activity and oxidative stress in the glioma microenvironment. Our findings revealed that D-mannose may be able to shift the host immune response toward tumor suppression and could be a potential new therapeutic direction for these difficult-to-treat tumors.

**Abstract:**

Host immune response in the tumor microenvironment plays key roles in tumorigenesis. We hypothesized that D-mannose, a simple sugar with anti-inflammatory properties, could decrease oxidative stress and slow glioma progression. Using a glioma stem cell model in immunocompetent mice, we induced gliomas in the brain and tracked MPO activity in vivo with and without D-mannose treatment. As expected, we found that D-mannose treatment decreased the number of MPO^+^ cells and slowed glioma progression compared to PBS-treated control animals with gliomas. Unexpectedly, instead of decreasing MPO activity, D-mannose increased MPO activity in vivo, revealing that D-mannose boosted the MPO activity per MPO^+^ cell. On the other hand, D-glucose had no effect on MPO activity. To better understand this effect, we examined the effect of D-mannose on bone marrow-derived myeloid cells. We found that D-mannose modulated MPO activity via two mechanisms: directly via N-glycosylation of MPO, which boosted the MPO activity of each molecule, and indirectly by increasing H_2_O_2_ production, the main substrate for MPO. This increased host immune response acted to reduce tumor size, suggesting that increasing MPO activity such as through D-mannose administration may be a potential new therapeutic direction for glioma treatment.

## 1. Introduction

Glioblastoma (GBM) is the most malignant subtype of high-grade glioma. It is composed of highly malignant cells that can exhibit widespread infiltration into adjacent and distant brain regions. Recent studies strongly suggest that the immune system has both positive and negative effects on tumorigenesis and that the glioma immune microenvironment is an essential component for tumorigenesis. The glioma microenvironment is highly immunosuppressive, thereby inhibiting the efficiency of immune treatments [1]. As such, currently, there is no effective immunotherapy against GBM, and multi-modality treatment with surgery, radiotherapy, and chemotherapy provides a median survival of only 18 months [2,3].

Tumor-related inflammation can lead to the development of vascular growth and tissue remodeling in part through hypoxia and oxidative stress [4,5]. As such, reactive oxygen species (ROS) are potential therapeutic targets in manipulating the tumor immune microenvironment and in improving outcome [6]. The most important source of reactive oxygen species and oxidative stress is the isoforms of nicotinamide adenine dinucleotide phosphate dehydrogenase (NADPH) oxidase. NADPH oxidases are a family of transmembrane proteins and include oxygen- and NADPH-dependent oxidoreductases that produce H_2_O_2_ in various cell types and tissues, often in response to growth factor and immune mediators [7]. Myeloperoxidase (MPO) is another oxidant-producing enzyme linked to both inflammation and NADPH oxidase activity [8]. MPO is a glycosylated heme-enzyme present in the azurophilic granules of myeloid cells (neutrophils, microglia, and macrophages). MPO catalyzes the reaction between chloride and hydrogen peroxide to generate the potent oxidant hypochlorous acid (HOCI) and other reactive oxygen/nitrogen species. MPO has been found in mature macrophages in atherosclerosis [9], in brain tissue showing Alzheimer-type pathology [10] or Parkinson’s disease [11], and in multiple sclerosis lesions [12], but it is also released in the tumor immune microenvironment [13]. Thus, MPO and MPO-mediated products likely play a role in the glioma immune microenvironment. 

Mannose is a component of glycoproteins. It is generated by phosphomannose isomerase from the glycolytic intermediate fructose-6-phosphate, and its availability is dependent on glycolytic flux. Mannose is essential for proper protein glycosylation. Interestingly, it has been suggested that deglycosylation of MPO could decrease MPO’s enzymatic activity [14]. In addition, D-mannose has been reported to be a safe and selective therapy in the treatment of urinary tract infections due to its anti-adhesive effect and, in peripheral tumors, due to the accumulation of mannose-6-phosphate inside the cancer cells that could impair tumor cell metabolism [15,16,17]. As the immune microenvironment is important in many therapeutic strategies and D-mannose can affect immune cell function [18,19], we aimed to investigate the effects that D-mannose has on the oxidative immune response in the glioma microenvironment.

## 2. Materials and Methods

### 2.1. Tumor Cell Cultures

The CT-2A-luc cells were generously donated by Dr. Martha R. Neagu. The cells were incubated at 37 °C with humidified air containing 5% CO_2_. Monolayer CT-2A-luc cells were cultured in Dulbecco’s modified eagle medium with high glucose (DMEM; Thermo Fisher Scientific, Grand Island, NY, USA) supplemented with 10% fetal bovine serum (FBS; Sigma-Aldrich, St. Louis, MO, USA) and 1% penicillin-streptomycin. To generate neurospheres, CT-2A monolayer cells were enzymatically dissociated by accutase (Stem Cell Technology, San Diego, CA, USA) and seeded in 25 cm^2^ culture dishes at the cell concentration of 1 × 10^5^ cells/mL in serum-free medium, composed of advanced DMEM/F12 medium (Life Technologies, Carlsbad, CA, USA) with l-glutamine (2 mM; Cellgro, Manassas, VA, USA), 1% N2 supplement (Life Technologies), 1% penicillin-streptomycin (Cellgro), recombinant EGF (20 ng/mL; R&D Systems, Minneapolis, MN, USA), and recombinant FGF2 (20 ng/mL; Peprotech, East Windsor, NJ, USA). After 10–11 days, the neurospheres were collected, enzymatically dissociated with accutase (Stem Cell Technology), and prepared for further studies [20,21].

### 2.2. In Vivo Animal Study

All animal experiments were approved by the subcommittee on research animal care at the Massachusetts General Hospital. Eight- to nine-week-old C57BL/6J female mice were obtained from Jackson laboratory. Dissociated NS/CT-2A-luc cells (7–8 × 10^4^) were implanted stereotactically into the brain (2.5 mm lateral and 1 mm anterior to Bregma and 3 mm deep) to generate an orthotopic intracranial tumor [22]. Three days after tumor implantation, mice were randomly divided into two groups and intraperitoneally injected with either D-mannose (450 mg/kg; Sigma-Aldrich) or PBS as the control [23]. The mice then were monitored daily for signs of discomfort or neurological symptoms.

### 2.3. In Vivo Bioluminescence Imaging

Mice were injected intraperitoneally with 150 mg/kg D-luciferin in PBS 10 min prior to imaging. Imaging was performed using AMI HTX (Spectral Instruments Imaging, Tucson, AZ, USA). The mice were anesthetized with isoflurane prior and during the imaging (*n* = 9–10 mice per group). Total photon flux (photon/s) was measured from a fixed region-of-interest (ROI) over the skull using BLI and XQuartz software (version 2.7.11).

### 2.4. In Vivo MR Imaging

D-mannose-treated and control (PBS-treated) mice were anesthetized with isoflurane and imaged used a 4.7 Tesla (Bruker, Billerica, MA, USA) MRI scanner with a dedicated mouse brain coil. T_1_-weighted MRI images were acquired before and 1 h after the intravenous administration of 0.3 mmol/kg of MPO-Gd using rapid acquisition with refocused echo (RARE) sequences (repetition time (TR): 873 ms, slice thickness: 0.6 mm, echo time (TE): 12.77 ms, field of view (FOV): 100, and matrix size (MTX): 192 × 192). T2-weighted imaging (repetition time (TR): 4000 ms, slice thickness: 0.6 mm, echo time (TE): 40 ms, field of view (FOV): 100, and matrix size (MTX): 192 × 192) was also acquired. Regions of interest (ROI) including peritumoral area, contralateral brain tissue, and background were selected using the Horos software version v3.3.6. Contrast-to-noise-ratio (CNRs) were computed for each ROI with the formula: CNR (postcontrast ROI_lesion_ − postcontrast ROI_contralateral brain_)/SD_noise_ − (precontrast ROI_lesion_ − precontrast ROI_contralateral brain_)/SD_noise_, where ROI_lesion_ is peritumoral enhancing area and SD_noise_ is the standard deviation of noise measured from an ROI placed in an empty area of the image (*n* = 3 mice per group). Tumor volumes were calculated by summation of the tumor areas on all T2-weighted slides using Horos software version v3.3.6 (*n* = 8–9 mice per group).

### 2.5. Flow Cytometry Analysis 

To analyze the tumor inflammatory immune cells, brains were rapidly removed 21–28 days after tumor implantation and stored in 10 mL of ice-cold PBS (*n* = 9–10 mice per group). Then, the brains were mechanically disrupted with a glass homogenizer and passed through a 40 µm nylon cell strainer (BD Biosciences, San Jose, CA, USA), and single cells were isolated from 30/70 Percoll (GE Healthcare, Boston, MA, USA) gradient interface. For surface staining anti-CD16/CD32, anti-CD11b APC/CY7 were obtained from Biolegend (San Diego, CA, USA), and for intra cellular staining, anti-MPO-biotin was purchased from Hycult (Plymouth Meeting, PA, USA). Cells were spun, counted, and resuspended in FACS buffer and incubated first with anti-CD16/CD32 to block Fc binding site for 20 min and then washed with washing buffer for 3 times. Cells were then incubated with antibody against surface markers for 30 min at 4 °C in the dark. For intracellular staining, the cells were then fixed and permeabilized using 1× Fix/Perm solution (BD Bioscience), washed in 1× permeabilization buffer (BD Bioscience), and stained with anti-MPO-biotin for 30 min at 4 °C in the dark, and then, we used streptavidin-conjugated brilliant violet 605 secondary from Biolegend (San Diego, CA, USA) to label biotinylated anti-MPO. The cells were subsequently washed and resuspended in FCS buffer. All leukocytes were identified as CD11b^+^ cells. These cells were then further identified as MPO^+^ and MPO^−^ cells. Data were acquired on LSRII flow cytometer (BD Bioscience) and analyzed with BD FlowJo software (version 10.4).

### 2.6. Leukocyte Isolation and Stimulation

Mouse leukocytes were isolated from mouse bone marrow as follows. Mice were sacrificed, and femur and tibias were removed. The epiphysis was cut off and bone marrow was flushed out with a 25-gauge needle and syringe containing PBS supplemented with 20 mM HEPES and 0.5% FCS buffer. To create single cell suspensions, bone marrow was filtered through a 40 µm cell strainer (BD Biosciences, San Jose, CA, USA) and spun at 350 g at room temperature for 6 min, and the supernatant was discarded. RBCs were lysed with the RBC lysis buffer (Biolegend, San Diego, CA, USA) as per the manufacturer’s instruction. The cell pellet was resuspended in 5% FCS buffer and immediately used in experiments. Then, to trigger degranulation, isolated cells were kept in suspension at a concentration of 6 × 10^6^ cells/mL and preincubated with different concentrations of D-mannose (1 mg/mL, 2 mg/mL, and 4 mg/mL) for 10 min followed by incubation with 1 µg/mL phorbol 12-myristate 13-acetate (PMA) (Sigma) for 2 h at room temperature (*n* = 3 for each condition). We also preincubated cells with D-glucose at a concentration of 2 mg/mL for comparison. To evaluate the effect of glycolysis inhibition on MPO activity, cells were incubated with 2 mg/mL of 2-deoxy-D-glucose (2DG) (Sigma) for 10 min followed by incubating with 2 mg/mL D-mannose, and then, cells were incubated with PMA as described above. Supernatant was then harvested for MPO ELISA and MPO activity assays. The assays were performed immediately after adding 50 µL of 100 mM ADHP (10-acetyl-3,7-dihydroxyphenoxazine, AAT Bioquest, Sunnyvale, CA, USA) solution and measured at the excitation wavelength of 535 nm and emission wavelength of 590 nm. The data were collected from a total of 13 kinetic cycles. H_2_O_2_ production was measured by incubating the samples with horseradish peroxidase (HRP) (Sigma) and 3,3′,5,5′-tetramethylbenzadine (TMB) (Sigma) for 20 min, and then, the stop solution was added. The absorbance was measured at 570 nm. The data were normalized to untreated cells.

### 2.7. MPO ELISA and Activity Analysis

Brains were homogenized separately in 500 µL of cetyltrimethylammonium buffer (50 mM potassium phosphate at pH 6.0 with 50 mM CTAB) by a tissuemiser homogenizer (Fisher Scientific, Waltham, MA, USA). The samples were then sonicated for 30 s and centrifuged at 13,000 rpm for 15 min. The supernatant was used for protein analysis with a BCA protein assay kit (Thermo Scientific, Waltham, MA, USA). To specifically capture MPO, aliquots from a homogenized brain supernatant were incubated with MPO-antibody (Hycult) precoated ELISA plates for 1 h at RT. Assay wells were then washed 3 times with washing buffer (PBS with 0.05% Tween20) and once with only PBS. The antibody-captured MPO activity was measured after adding 49 µL of PBS and 1 µL of 1:100 diluted 3% hydrogen peroxidase to each well followed by 50 µL of 100 mM ADHP solution [24]. 

### 2.8. Statistical Analysis

The results are presented as mean ± standard error of measurement (SEM). For inter-group comparison of in vivo and ex vivo experiments, the Mann–Whitney test was used. The inter-group differences of in vitro D-mannose effect on MPO activity were analyzed using the Kruskal–Wallis test. For comparisons between multiple conditions of the in vitro experiments, ordinary one-way ANOVA was used. *p*-values < 0.05 were considered statistically significant. Data were analyzed using Graphpad Prism software 8 (GraphPad Prism, Inc., La Jolla, CA, USA).

## 3. Results

### 3.1. Bioluminescence (BLI) and MRI Imaging Revealed D-Mannose Treatment Slows Glioma Growth

**Figure 1:**
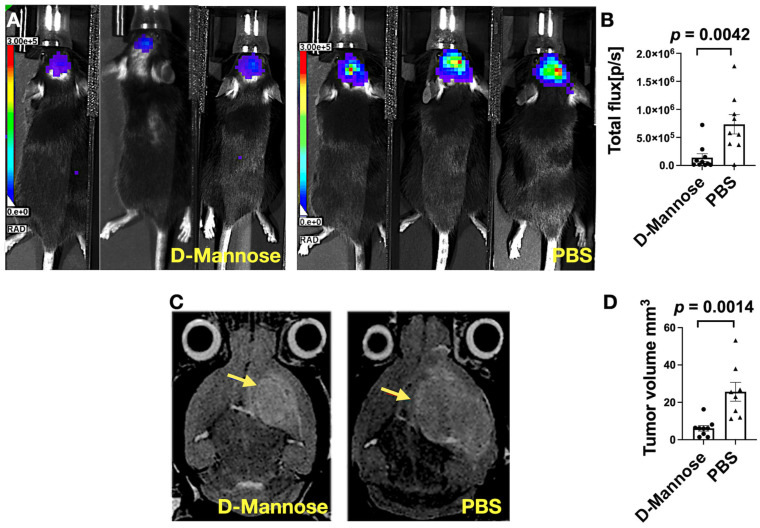
Tumor size characterized by bioluminescence (BLI) and T2-weighted MR imaging. (**A**) Representative BLI images performed at the third week after glioma induction for the D-mannose-treated and PBS-treated groups. (**B**) Quantification of tumor sizes on BLI (*n* = 8–9 per group). (**C**) Representative T2-weighted MRI images performed at the fourth week after glioma induction for the two groups. Arrows indicate the tumors. (**D**) Quantification of tumor sizes (*n* = 8–9 per group). Data are shown as mean ± standard errors of measurements.

To assess the effect of D-mannose treatment on tumor size in vivo, we imaged the mice using bioluminescence three weeks after tumor implantation. We found that the mean light intensity was significantly lower in the brains of the D-mannose-treated group than those in the PBS-treated group (Figure 1A,B; *p* = 0.0042), demonstrating the presence of fewer luciferase-expressing glioma cells after D-mannose treatment. We then used T2-weighted MR imaging to measure lesion size (tumor and associated parenchymal abnormalities) four weeks after tumor induction, which showed smaller lesions in the D-mannose-treated group than those in the PBS-treated mice (Figure 1C). Quantification of lesion volumes confirmed the imaging findings (Figure 1D; *p* = 0.0014). 

### 3.2. D-Mannose Increases MPO Amount and Activity In Vivo

**Figure 2:**
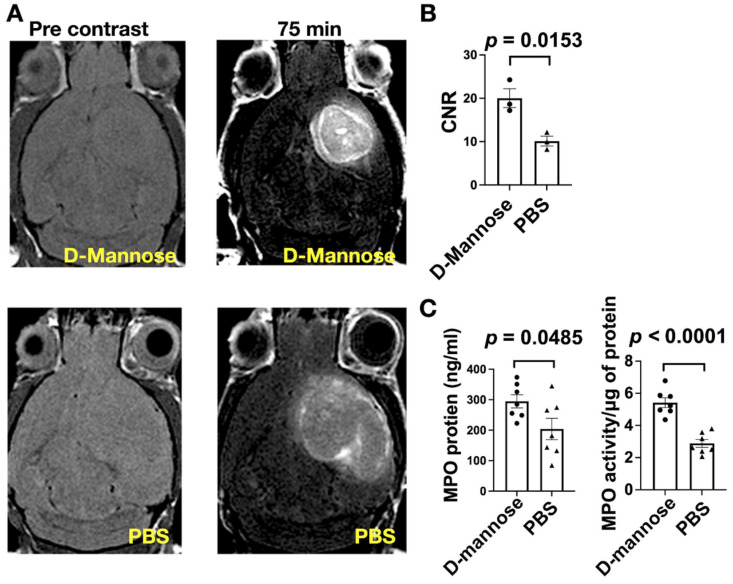
MPO-Gd MR imaging, and MPO protein level and activity in glioma mice. (**A**) Representative MRI of glioma mice performed before and at 75 min after MPO-Gd administration (*n* = 3 per group). (**B**) Comparison of contrast-to-noise ratios (CNR) in animals with D-mannose treatment and control group. (**C**) Ex vivo measurements of MPO protein level and MPO activity from brain tissues from glioma-bearing mice (*n* = 7 per group). Data are shown as mean ± SEM.

To better understand how D-mannose treatment affected inflammatory response in the tumor microenvironment, we performed T1-weighted MR imaging with the MPO-activatable MRI agent MPO-Gd, which has been demonstrated to be specific and sensitive to extracellular MPO activity in multiple inflammatory and tumor models [25,26,27,28,29]. Mice were imaged during the fourth week after induction (Figure 2A). Surprisingly, MPO-Gd signal (expressed as CNR) in D-mannose-treated mice showed a twofold increase compared to that of the control group (Figure 2B, *p* = 0.0153), signifying that D-mannose administration increased MPO activity in vivo. To validate that the imaging results indeed represented MPO activity, we collected brain samples from both groups after imaging to measure MPO protein and activity ex vivo. Consistent with MR imaging results, in the D-mannose-treated group, both MPO protein and MPO activity were elevated compared to those of the control group (Figure 2C, *p* = 0.0.0485 for MPO protein and *p* < 0.0001 for MPO activity). 

**Figure 3:**
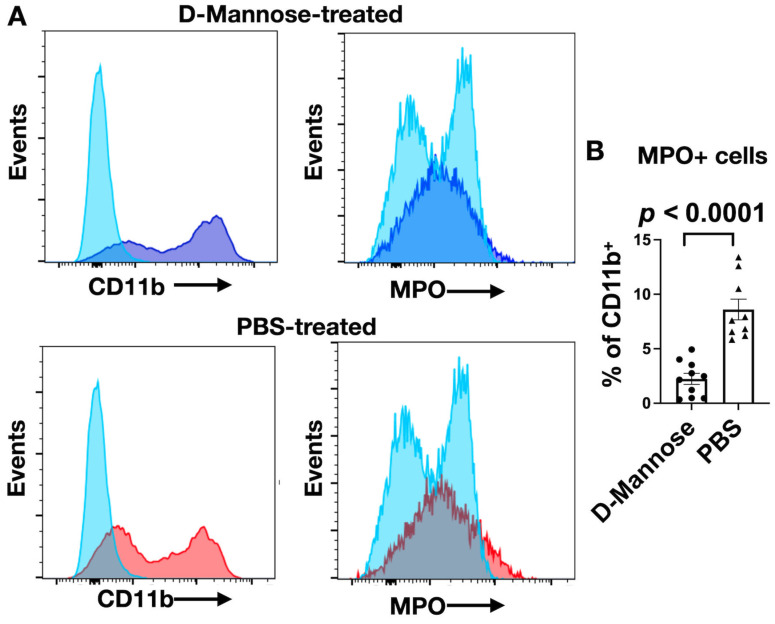
Decrease in MPO^+^ cells in brains of D-mannose-treated mice. (**A**) Representative flow cytometry histograms comparing the number of MPO^+^ cells between D-mannose-treated and PBS-control group (light blue: negative control (unstained), dark blue: D-mannose-treated, and red: PBS-treated). (**B**) Quantification of the number of MPO^+^ cells as a percentage of CD11b^+^ leukocytes (*n* = 9–10 mice per group). Data are shown as mean ± SEM.

However, contrary to the increased extracellular MPO activity, MPO^+^ cells (Figure 3A) were less abundant in the D-mannose-treated group than in the PBS-treated group (Figure 3B, *p* < 0.0001). Taken together, these results revealed that D-mannose treatment decreased the number of pro-inflammatory cells recruited to the glioma microenvironment, but each of these cells secreted more active MPO. 

### 3.3. D-Mannose Increases MPO Activity In Vitro

**Figure 4:**
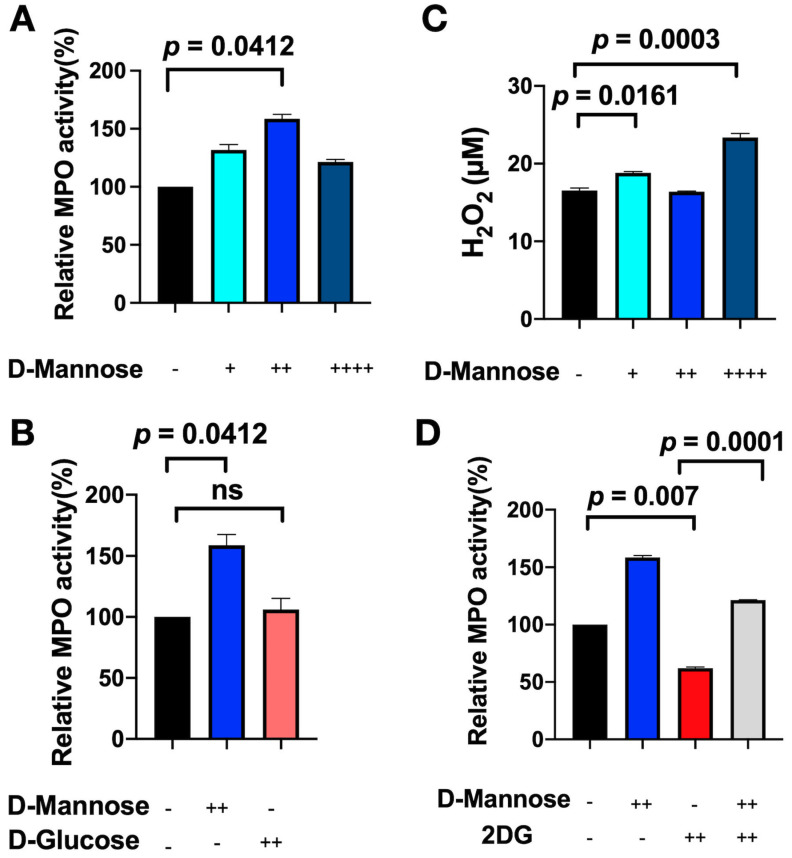
Biochemical analysis of PMA-stimulated mouse bone marrow-derived leukocytes. (**A**) Relative MPO activities were normalized to untreated leukocytes samples (+ = 1 mg/mL, ++ = 2 mg/mL, ++++ = 4 mg/mL). (**B**) Comparison of MPO activity with and without D-mannose and D-glucose (++ = 2 mg/mL). ns = not significant. (**C**) The amount of H_2_O_2_ produced by leukocytes was measured under similar conditions as in (**A**). (**D**) Comparison of MPO activity produced by leukocytes with and without D-mannose and 2-deoxy-D-glucose (2DG). All data are in mean ± SEM.

To determine a possible mechanism for how D-mannose modulates MPO activity in leukocytes, we treated bone-marrow-derived leukocytes with different concentrations of D-mannose. We found that the media of D-mannose-treated leukocytes contained higher MPO activity than those of untreated cells (Figure 4A, *p* = 0.0412). Furthermore, our results showed that there was an inverse parabolic relationship between D-mannose and MPO activity; 2 mg/mL of D-mannose was found to be optimal. Too little or too much D-mannose led to decreased MPO activity (Figure 4A). However, when cells were treated with D-glucose, no similar effect on MPO activity was observed (Figure 4B). We next determined if D-mannose could affect H_2_O_2_ production. We found that D-mannose increased the amount of H_2_O_2_ produced in D-mannose-treated cells compared to untreated cells at low and high concentrations but decreased H_2_O_2_ production at the intermediate concentration (Figure 4C, *p* = 0.0161 for 1 mg/mL of D-mannose and *p* = 0.0003 for 4 mg/mL of D-mannose). These results appeared to inversely be correlated with MPO activity (Figure 3A), suggesting that D-mannose’s effect on MPO activity could be in part related to its effect on H_2_O_2_ production. 

We also investigated whether D-mannose can increase MPO activity through N-linked protein glycosylation. We treated the cells with 2DG, which can inhibit glycolysis and N-glycosylation of proteins. We found that the medium of 2DG-treated cells demonstrated lower MPO activity compared to that of untreated cells (Figure 4D, *p* = 0.007). Next, we treated leukocytes with both 2DG and D-mannose. We found that 2DG-D-mannose-treated cells secreted significantly higher MPO activity compared to 2DG-treated cells (Figure 4D, *p* = 0.0001). These results revealed that the inhibitory effect of 2DG on N-linked glycosylation can be rescued by the addition of D-mannose.

## 4. Discussion

In this study, we found that D-mannose markedly slowed tumor growth in a mouse glioma model. It has anti-inflammatory properties and can block signaling related to MPO [30]. As such, we expected D-mannose treatment to decrease MPO activity in the glioma microenvironment. Instead, we found that D-mannose *increased* MPO activity in the glioma microenvironment. Despite the increase in total MPO activity, there were fewer MPO^+^ cells found in the glioma microenvironment, suggesting that each MPO^+^ cell secretes more active MPO. In vitro experiments corroborated the increased MPO activity secreted by D-mannose-treated myeloid cells. We identified two mechanisms by which D-mannose can modulate MPO activity: (1) indirectly by modulating the production of H_2_O_2_ and (2) directly by enhancing N-linked glycosylation of MPO. Our results showed that this elevated MPO activity after D-mannose treatment suppressed tumor growth. 

ROS play an important role in the tumor immune microenvironment. MPO is a heme-containing enzyme that, in the presences of H_2_O_2_ and halides, catalyzes the formation of reactive oxygen intermediates. Therefore, it mediates several oxidative and cytotoxic reactions at the site of inflammation. Our results revealed that D-mannose can modulate the production of H_2_O_2_ to indirectly impact MPO activity. However, the effect is not a direct linear response. Instead, we found an inverted parabolic curve where too little or too much H_2_O_2_ led to a decrease in MPO activity. This is likely due to the fact that a low amount of H_2_O_2_ would not provide sufficient substrates for MPO, while _a_ high amount of H_2_O_2_ can disrupt MPO activity [31,32]. Based on this, the in vivo dose of D-mannose, which was derived from prior studies that empirically determined the optimal dose [23], likely generates an amount of H_2_O_2_ that is near the peak of the curve (Figure 3A). Future experiments should take this parabolic response into account to further optimize the dose for different species, especially if translated to human.

N-glycosylation is an enzymatic process by which glycosidic linkages are formed between saccharides and proteins [33]. Previous studies suggested that the rigid heme architecture is essential for enzyme activity of peroxidases, and glycans are closely configured around the heme pocket. The change in glycans can modify the overall protein structure, alter the heme pocket, and affect interaction of MPO with its substrates [34,35]. Furthermore, it has been demonstrated that the loss of the glycan terminus decreased MPO’s enzymatic activity [14,36]. 2DG is commonly used as a glycolysis inhibitor and interferes with N-linked glycosylation predominately by competition with D-mannose. Indeed, we found that D-mannose increased MPO activity while 2DG suppressed MPO activity, and D-mannose can partially overcome the suppressive effect from 2DG. These findings revealed that D-mannose enhances N-linked glycosylation to increase MPO activity. As MPO is normally a highly oxidizing, deleterious enzyme, such an upregulation of MPO activity is potentially also harmful to the host in addition to the glioma. However, this increase appeared to be tempered by limiting this response to the glioma microenvironment and that there were fewer number of MPO^+^ cells, likely due to the effect D-mannose has on MPO signaling [30]. 

Interestingly, a prior study reported that, in a toxin-induced lung cancer model, MPO inhibition decreased tumor multiplicity [37]. In this study, we found that increasing MPO activity decreased glioma size. Besides differences in organs and models that have different immune environments, chronic low levels of MPO activity could serve to induce mutagenesis and promote tumorigenesis, especially during early tumor development. In that environment, decreasing MPO activity would be beneficial. Indeed, in the lung cancer study, the authors found that inhibiting the MPO activity did not have an effect on implanted tumors, suggesting that MPO activity has a role in the early phase of cancer development. On the other hand, when MPO activity is acutely increased in established tumors, as was the case in our study with implanted glioma treated with D-mannose, the increased acute inflammatory response becomes tumoricidal. Future studies could be designed to further elucidate these different roles of MPO and ways to better manipulation MPO activity to improve outcome.

This study had several limitations. D-mannose is readily available as a dietary supplement. Thus, in patients, the expected route of administration is likely to be oral in the form of a pill or capsule. In patients with urinary tract infections (UTIs), the oral clinical dose of D-mannose is 2 g once to twice daily [16]. Assuming a patient weighs 60 kg, this dose translates to about 33 mg/kg. Our mouse dose is 450 mg/kg, and with a conversion factor of 12.3 [38], the human equivalent dose is 36.6 mg/kg, which is similar to the human dose used in UTI. However, in this study, we opted to administered D-mannose intraperitoneally instead of via oral gavage because a gavage may be too stressful for these glioma-bearing animals. As such, the bioavailability may be different, though a recent pharmacokinetic study showed that 90% of ingested D-mannose is rapidly absorbed into the blood, suggesting that oral and parenteral administration may have similar bioavailability [39]. Given the inverted parabolic response of D-mannose to MPO activity, a careful dose-finding study as well as studies identifying the distribution of dietary D-mannose in the CNS will be needed for translation. In addition, the favorable anti-glioma effect should be further studied and confirmed in different models and conditions prior to potential translation to patients.

## 5. Conclusions

We demonstrated that D-mannose unexpectedly increased MPO activity in glioma, which markedly slowed glioma growth in a mouse model. We identified direct and indirect mechanisms by which D-mannose can modulate MPO activity in the glioma microenvironment. Our findings provided mechanistic insight into the role that MPO and oxidative stress play in glioma growth and support future investigations of D-mannose as a potential low-cost treatment option for this devastating disease. 

## Data Availability

The data presented in this study are available within the article.
